# Trends in burnout and psychological distress in hospital staff over 12 months of the COVID-19 pandemic: a prospective longitudinal survey

**DOI:** 10.1186/s12995-022-00352-4

**Published:** 2022-05-25

**Authors:** Robert G. Maunder, Natalie D. Heeney, Jonathan J. Hunter, Gillian Strudwick, Lianne P. Jeffs, Leanne Ginty, Jennie Johnstone, Alex Kiss, Carla A. Loftus, Lesley A. Wiesenfeld

**Affiliations:** 1 Sinai Health, Room 915, 600 University Ave, Toronto, Ontario M5G 1X5 Canada; 2 Department of Psychiatry, University of Toronto, Toronto, Canada; 3 Centre for Addiction and Mental Health, Toronto, Canada; 4 Institute of Health Policy, Management and Evaluation, University of Toronto, Toronto, Canada; 5 Lunenfeld-Tanenbaum Research Institute, Sinai Health, Toronto, Canada; 6 Department of Laboratory Medicine and Pathobiology, University of Toronto, Toronto, Canada; 7 Department of Research Design and Biostatistics, Sunnybrook Research Institute, Toronto, Canada; 8 Dalla Lana School of Public Health, University of Toronto, Toronto, Canada

**Keywords:** COVID-19, Pandemic, Healthcare workers, Nurses, Burnout, Resilience, Psychological distress

## Abstract

**Background:**

The mental health effects of healthcare work during the COVID-19 pandemic have been substantial, but it is not known how long they will persist. This study aimed to determine if hospital workers’ burnout and psychological distress increased monotonically over 1 year, during which waves of case numbers and hospitalizations waxed and waned, or followed some other pattern.

**Methods:**

A prospective longitudinal survey was conducted at four time-points over 1 year in a cohort of 538 hospital workers and learners, which included validated measures of burnout (emotional exhaustion scale of Maslach Burnout Inventory) and psychological distress (K6). Repeated measures ANOVA tested changes over time and differences between subjects by occupational role, age and ethnic group. The direction and magnitude of changes over time were investigated by plotting rates of high scores (using cut-offs) at each time-point compared to case rates of COVID-19 in the city in which the study took place.

**Results:**

There were significant effects of occupational role (F = 11.2, *p* < .001) and age (F = 12.8, *p* < .001) on emotional exhaustion. The rate of high burnout was highest in nurses, followed by other healthcare professionals, other clinical staff, and lowest in non-clinical staff. Peak rates of high burnout occurred at the second or third measurement point for each occupational group, with lower rates at the fourth measurement point. Similarly to the results for emotional exhaustion, rates of high psychological distress peaked at the spring 2021 measurement point for most occupational groups and were higher in nurses than in other healthcare professionals.

**Conclusions:**

Neither emotional exhaustion nor psychological distress was rising monotonically. Burnout and psychological distress were consistently related to occupational role and were highest in nurses. Although emotional exhaustion improved as the case rate of COVID-19 decreased, rates of high emotional exhaustion in nurses and other healthcare professionals remained higher than was typically measured in hospital-based healthcare workers prior to the pandemic. Ongoing monitoring of healthcare workers’ mental health is warranted. Organizational and individual interventions to support healthcare workers continue to be important.

## Background

A global pandemic of COVID-19 was declared by the World Health Organization on March 11, 2020 [1]. New cases have occurred in waves lasting months, which vary by geographic region [2]. The mental health effects of working in healthcare during the pandemic are widely appreciated to be substantial, resulting from uncertainty, risk of infection, high volumes of acutely ill patients, and long hours, among other factors. Reports from cross-sectional studies have consistently indicated, for example, high rates of depressive and anxiety symptoms, sleep disturbance and burnout in healthcare workers [3–6].

Professional burnout is a particularly important outcome of occupational stress in healthcare because, in addition to its impact on individual professionals, burnout has adverse consequences for the healthcare system and patients, including absenteeism, higher workforce turnover, reduced productivity, increased medical errors, and reduced quality of care [7–10]. As described by Maslach and colleagues [11], burnout is comprised of three components: emotional exhaustion, depersonalization (becoming indifferent or emotionally distant), and a diminished sense of personal accomplishment. Burnout was recognized prior to the COVID-19 pandemic to be a major occupational risk in health care [12, 13]. Workplace factors that were known to contribute to burnout include high workload, lack of scheduling flexibility, the burden of administrative tasks, and concern about workplace safety [14–20]. In addition to workplace stressors that were present prior to the COVID-19 pandemic, additional potential sources of burnout have emerged, including constraints on care that cause moral distress [21, 22], and redeployment to unfamiliar types of work [23].

While burnout is not considered to be a mental illness, symptoms of common mental disorders have also been measured to determine the impact of the COVID-19 pandemic on healthcare workers’ mental health. A systematic review containing 65 studies that include 97,333 healthcare workers from 21 countries found pooled prevalence’s of 21.7, 22.1, and 21.5% for moderate depression, anxiety, and post-traumatic stress disorder respectively during COVID-19 [24]. Similarly, in a systematic review and meta-analysis including 29 studies and 22,380 hospital staff caring for COVID-19 patients from countries in Europe and Asia, the prevalence of depression, anxiety, and stress was 24.3, 25.8, and 45%, respectively [6].

Regarding which healthcare workers have been at greatest risk of adverse psychological outcomes during the COVID-19 pandemic, a meta-analysis of studies published up to July 2020 indicated rates of distress and burnout were higher in nurses, females and frontline workers than in doctors, males and non-frontline workers [25]. Some studies published since this meta-analysis have also indicated higher rates in nurses than doctors although this finding is not universal [21, 26, 27].

The long-term impact of the COVID-19 pandemic on healthcare workers’ mental health is not known. Some factors suggest that the mental impact of the pandemic will be enduring. In particular, after the 2003 outbreak of severe acute respiratory syndrome (SARS) had resolved, healthcare workers in affected hospitals reported a range of stress effects that continued to be significantly elevated compared to workers in non-affected hospitals for as long as they were followed, which was 18–24 months [28]. Of concern, the COVID-19 pandemic is far more severe, widespread and persistent than the SARS outbreak and has severely affected many aspects of life in the community as well as in the hospital, which suggests its mental impact will be greater, and may resolve more slowly. Furthermore, pre-pandemic rates of burnout in many settings were high, in the range of 20–40% [29–31], indicating that even full resolution of pandemic-related factors would still leave healthcare workers at considerable risk. On the other hand, there is much evidence that in general, the most common long-term response to exposure to highly aversive events is resilience [32].

As evidence emerges, prediction of future negative mental health consequences of the COVID-19 pandemic for healthcare workers will benefit from determining longitudinal trends in burnout and psychological distress among hospital workers. In particular, it would be useful to determine if negative mental health effects are rising monotonically over time, as one would expect to result from accumulating stress effects, or following a different pattern, possibly rising when COVID-19 cases are higher and improving as COVID-19 case rates decrease. Going forward, interventions to support healthcare workers’ well-being could differ in these two scenarios. Longitudinal data published thus far in the COVID-19 pandemic gives little insight into these long-term patterns because it has covered periods early in the pandemic, typically with either two measurement points or over a period of only a few weeks. Studies of longer-term patterns of burnout and psychological distress later in the pandemic have not yet been published.

We report on two mental health measures, namely the emotional exhaustion component of burnout and psychological distress, collected in the same cohort of hospital personnel at four time-points (approximately quarterly) from the fall of 2020 to the summer of 2021. Our primary question is whether indicators of these mental health problems are (i) rising monotonically, or (ii) following some other pattern, such as rising and falling in synchrony with the local epidemiological waves of COVID-19 cases. Our secondary questions are whether this pattern differs between personnel with different occupational roles, what proportion of hospital personnel participating in this survey are reporting levels of emotional exhaustion and psychological distress that are considered high at each time point, and how the depersonalization and personal accomplishment dimensions of burnout change over this period.

## Methods

### Study design and participants

A survey of the psychological well-being of a cohort of hospital staff, learners (nursing students, medical students, residents), and volunteers during the pandemic was conducted at two sites of Sinai Health (an urban acute care teaching hospital and a rehabilitation hospital, with > 6000 employed staff) in Toronto, Canada at four time-points. The survey methods have been described previously [33]. Briefly, the first survey (T1, “fall 2020”) was conducted from Sept 21-Nov 15, 2020. The first survey was open to all hospital employees, physicians, learners, volunteers, retail employees, and contractors. Potential participants were informed of an online survey link via “all-staff” emails (e.g. communication updates or newsletters) from the hospital or from their chiefs and directors, as well as information posters in high-traffic areas of the hospital. All surveys were completed online using software that is compliant with privacy standards (the Personal Health Information Protection Act, in this jurisdiction) (Alchemer, Louiseville, CO). Of 884 respondents who provided consent in a pre-survey recruitment phase, 538 (61.0%) completed a T1 survey to form the cohort for further follow-up. The denominator for an overall participation rate (i.e. the number of staff who were sufficiently informed to make a decision to participate or not) is not known because the survey was open to all staff and advertised by various means, but individual invitations to participate were not used. The entire salaried workforce who might have participated was approximately 6000. Subsequent surveys of the T1 cohort were conducted in these time periods in 2021: Jan 25-Feb 15 (T2, “winter 2021”), Apr 26-May 16 (T3, “spring 2021”), and Jul 26-Aug 15 (T4, “summer 2021”). All members of the T1 cohort were invited to participate in all subsequent surveys, even if they were on leave or were not working at the hospital at the time of a survey. Each survey included measures of emotional exhaustion and psychological distress, among other measures. Participants were randomized to a shorter (Express) or longer (Enriched) version of the survey. Surveys varied in length over time due to instruments that were included at only some time-points. The total length of the Express survey varied from 45 to 83 items and the total length of the Enriched survey varied from 77 to 137 items. Among those who participated in the first survey, the participation rate at subsequent time points (the numerator calculated as the number of surveys returned that included a valid measure of emotional exhaustion, psychological distress, or both) was: T2 *N* = 485 (90% of T1 cohort), T3 *N* = 424 (79%), T4 *N* = 409 (76%). The numbers of participants who had left the organization at each survey time-point were: T1–9, T2–21, T3–18, T4–24. Given the small amount of attrition, these were not considered further in the analysis. A gift card (about US$15 value) was provided for each completed survey. The study was approved by the Sinai Health Research Ethics Board.

### Measures

Burnout was measured with the Maslach Burnout Inventory (MBI-HSS), which measures emotional exhaustion, depersonalization, and diminished personal accomplishment [34]. Various case definitions of burnout based on MBI-HSS scores have been used previously, which differ in the choice of cut-off scores, and various combinations of the three scales [29]. In order to allow comparison to a broad range of prior studies, we used the emotional exhaustion scale as our primary continuous measure of burnout, and defined burnout using a cut-off of ≥27, which is commonly used to identify high emotional exhaustion [29–31]. As secondary measures, to describe trends in other aspects of burnout we measured depersonalization and personal accomplishment in those participants randomly selected for the Enriched survey, using the most common cut-offs of depersonalization ≥10 and personal accomplishment are ≤33 when reporting case numbers [29]. In this cohort Cronbach’s alpha at the four time-points varied from .94 to .95.

Psychological distress is comprised of depressive and anxiety symptoms. Psychological distress was measured with the Kessler K6, which has 6 items scored from 0 to 4, yielding a range of 0–24. The K6 strongly discriminates between community cases and non-cases of psychiatric disorders diagnosed by structured interview and has acceptable sensitivity and specificity [35, 36]. A cut-off of ≥13 indicates likely serious mental illness [37]. In this cohort Cronbach’s alpha at the four time-points varied from .85 to .87.

### Analysis

At T1, participants were sorted into four categories of occupational role based on professional qualifications (i.e. classified as “professionals” if their job is regulated by a professional college, or equivalent) and whether they reported close patient contact (if they were within two metres of a patient for more than 15 minutes in the previous month). The four occupational role categories were nurses, other healthcare professionals (as listed in Table 1), other clinical staff (non-professionals who reported close patient contact), and non-clinical roles (non-professionals without close patient contact). Nurses were analyzed separately based on prior evidence that nurses experienced a greater burden of stress than other professionals during an outbreak of extraordinary infectious disease [28], and have during the COVID-19 pandemic [25].

**Table 1:** Characteristics of participants

		N (%)
Role type^a^	Nursing	134 (24.9)
	Other clinical professionals	156 (29.0)
	Other clinical personnel	90 (16.7)
	Non-clinical personnel	158 (29.4)
Gender	Female	422 (78.6)
(Missing 1)	Male	85 (15.8)
	Other/Prefer not to say	30 (5.6)
Highest education	High school	13 (2.4)
	College diploma	79 (14.7)
	Undergraduate degree	176 (32.7)
	Professional/Graduate degree	270 (50.2)
Ethnic group	African/Black	30 (5.6)
(Missing 1)	Asian	148 (27.6)
	South Asian	35 (6.5)
	European/White	278 (51.8)
	Hispanic	15 (2.8)
	Other/Mixed/Multiple	31 (5.8)
Marital status	Single	211 (39.2)
	Married/Common-law	306 (56.9)
	Divorced/Separated/Widowed	21 (3.9)

^a^Specific job types, in descending order of number of participants. Groups with two or fewer members not listed. Some roles appear in both clinical and non-clinical lists as determined by patient contact as described by participant. Other clinical professionals: Physician, resident, dietician, occupational therapist, social worker, physiotherapist, manager of clinical area, speech language pathologist, pharmacist, respiratory therapist, spiritual care practitioner. Other clinical positions: Administrative assistant, medical imaging technologist, assistant to physician/occupational therapist/physiotherapist, retail employee, porter, clinical research staff, volunteer. Non-clinical positions: Research scientist, research staff, laboratory technician, corporate and administrative staff, administrative assistant, volunteer, manager of non-clinical area, building services staff, clerk, laboratory technologist, housekeeper

Participant characteristics were summarized using descriptive statistics. Continuous measures were summarized using means and standard deviations (SD). Categorical measures were summarized using counts and percentages. In order to provide context for changes in case rates of burnout and high psychological distress over time, daily rates of new cases of COVID-19 diagnosed in Toronto and COVID-19-related hospitalizations during the period of this study were downloaded from Public Health Ontario [38].

Changes over time in emotional exhaustion and psychological distress, as continuous variables, by occupational role were analyzed using repeated measures ANOVA, excluding subjects with missing data at any timepoint. Age was included as a covariate because lower age/less experience have been identified as correlates of higher burnout [39, 40]. Ethnic group (categorized as Asian/South Asian; European/White; Other) was included as a covariate because of discrimination and stigma experienced by Asian people during the pandemic. Gender was not included because it is confounded with occupational role in hospital settings (in particular, in this cohort nurses were 84% female and other non-physician healthcare workers were 87% female). Setting within the hospital (e.g. emergency department, intensive care unit) could not be included in the analysis because (i) many staff members work in multiple settings, and (ii) there were frequent dynamic changes in work settings for many staff members related to having multiple roles or having reassigned duties (e.g. medical nurses re-assigned to intensive care for a period of time and then returning to the medical unit). Within-subjects effects are reported using a Greenhouse-Geisser correction when Mauchly’s test of sphericity indicated a significant deviation from the assumption of sphericity. All analyses were done with IBS SPSS Statistics 28 (Armonk, New York).

## Results

The characteristics of personnel in the cohort at T1 are described in Table 1.

### Emotional exhaustion

**Fig. 1:**
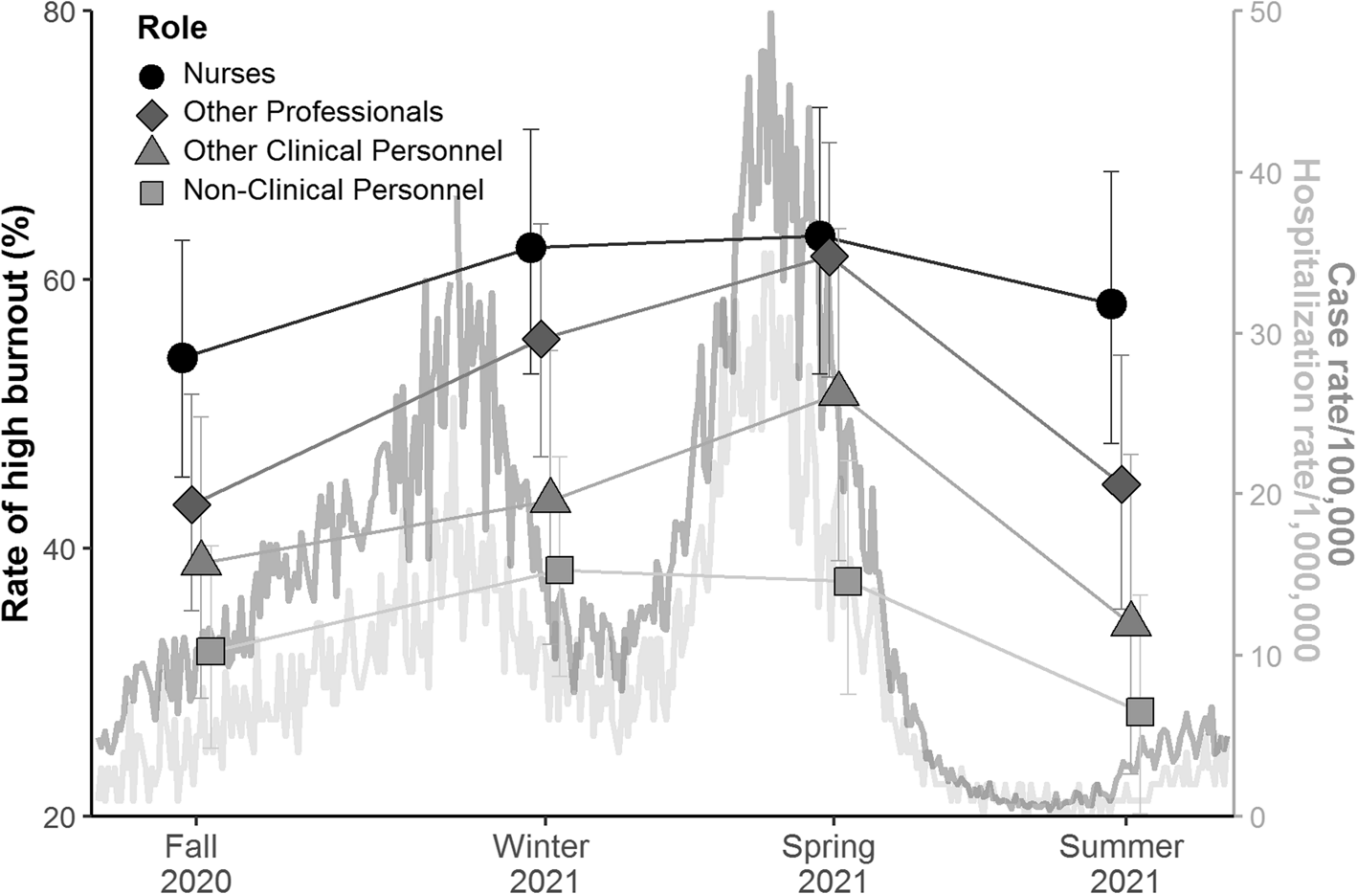
Case rates of high burnout by occupational role over 12 months in a Toronto hospital with daily case rates and hospitalization rates of COVID-19 in Toronto during the same period. Note: Figure shows proportion of each occupational group reporting high levels of the emotional exhaustion dimension of burnout, based on a cut-off of ≥ 27

Emotional exhaustion peaked at the winter 2021 or spring 2021 measurement point in each group and was lower by the summer 2021 measurement (Fig. 1). Repeated measures ANOVA of emotional exhaustion for participants for whom there were no missing data over four time-points (82 nurses, 102 other healthcare professionals, 60 other clinical staff, and 118 non-clinical staff) demonstrated significant between-subject effects of occupational role (F = 11.2, *p* < .001) and age (F = 12.8, *p* < .001), but not ethnic group (F = 1.3, *p* = .26). The interaction of occupational role X time was significant (F = 2.0, *p* = .04), but weak (estimated effect size, eta-squared = .02), indicating a modestly different pattern of change over time in occupational groups. Post hoc testing revealed that the relationship between age and emotional exhaustion was inverse (higher emotional exhaustion with younger age) and grew in strength over time (T1 R = −.06, *p* = .14, T2 R = −.09, *p* = .05, T3 R = −.19, *p* < .001, T4 R = −.21, *p* < .001). Regarding the direction and magnitude of differences in emotional exhaustion between staff with different occupational roles, the proportion of participants with high emotional exhaustion at each time point, by occupational role, is illustrated in Fig. 1 (with the epidemiological curves of case rates and hospitalization rates in Toronto included for context). The rate of high burnout was highest in nurses, followed by other healthcare professionals, other clinical staff, and lowest in non-clinical staff.

### Other burnout dimensions

Mean levels of depersonalization and personal accomplishment at each time point are provided in Table 2, as well as the proportion of participants whose depersonalization scores were high or personal accomplishment scores were low. Participants with high depersonalization scores were rising over time, while the numbers with low personal accomplishment scores were falling. The number of subjects randomly selected to complete these extra measures (the 50% of subjects assigned to the Enriched survey), who completed measures of depersonalization and personal accomplishment at all four time-points included too few participants in one particular occupation category (non-professional staff without patient contact, *N* = 8) to compare all occupational role groups. However, given the differences found for emotional exhaustion, we compared nurses (*N* = 41) to other healthcare professionals (*N* = 55). With respect to depersonalization there were significant differences between these groups, with nurses reporting greater depersonalization (F = 7.4, *p* = .008), with no significant effect of age. With respect to personal accomplishment there were also significant differences between nurses and other healthcare professionals (F = 9.6, *p* = .003), with nurses reporting lower personal accomplishment, with no significant effect of age.

**Table 2:** Depersonalization and personal accomplishment dimensions of attachment at four time-points

	Depersonalization				Personal accomplishment			
	N	mean	SD	High (%)	N	mean	SD	Low (%)
Fall 2020	277	4.80	5.59	18.1	270	34.42	10.99	37.0
Winter 2021	245	5.32	6.28	18.4	238	34.47	10.2	36.6
Spring 2021	154	6.42	6.86	23.4	148	37.85	7.05	20.3
Summer 2021	153	6.67	7.12	26.8	147	37.06	8.90	29.9

Depersonalization scores range from 0 to 30, with higher scores indicating greater burnout. High scores are based on a cut-off of ≥10. Personal accomplishment scores range from 0 to 48 with lower scores indicating greater burnout. Low scores are based on a cut-off of ≤33

### Psychological distress

**Fig. 2:**
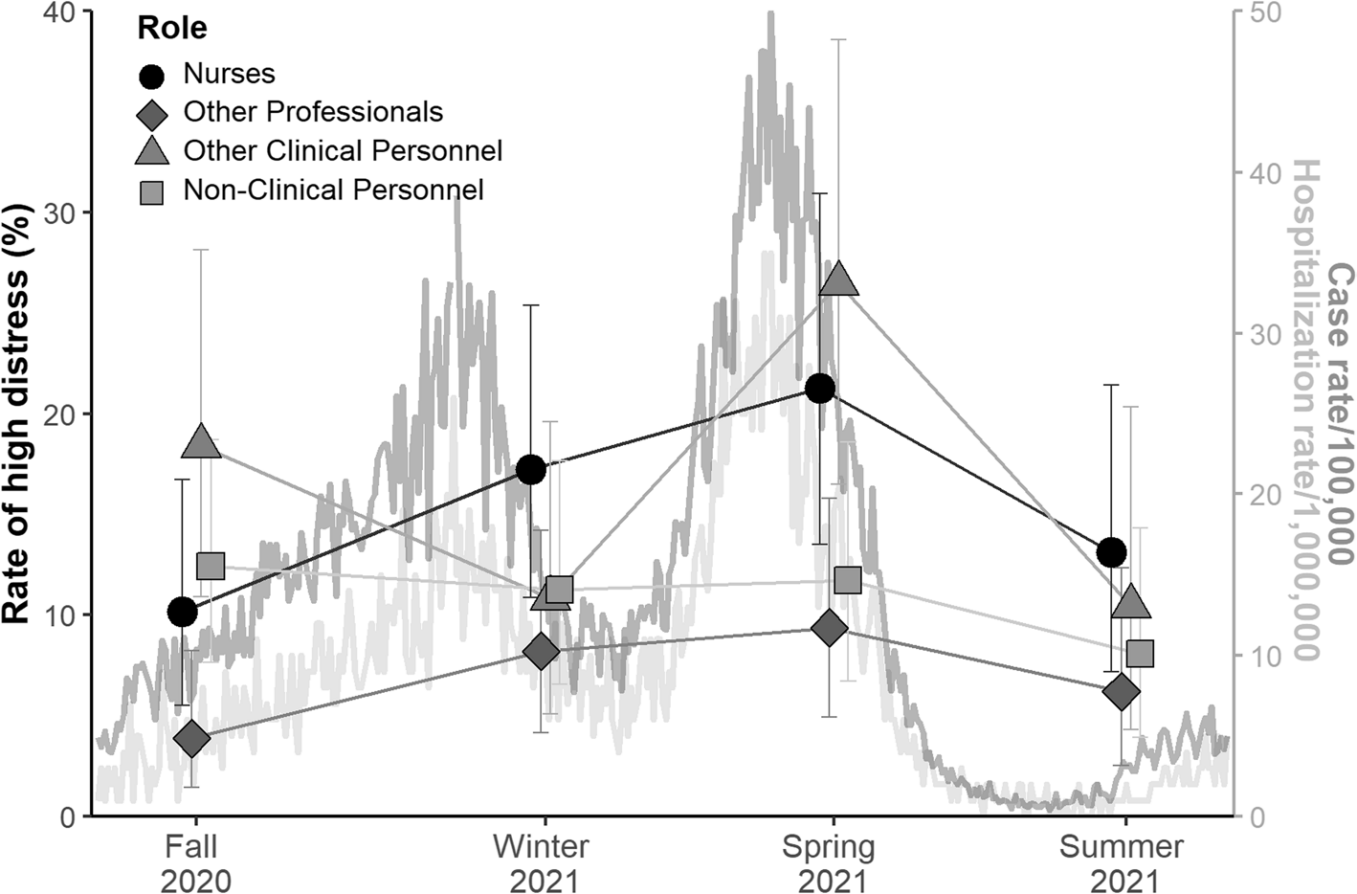
Case rates of high psychological distress by occupational role over 12 months in a Toronto hospital with daily case and hospitalization rates of COVID-19 in Toronto during the same period. Note: Figure shows proportion of each occupational group reporting high levels of psychological distress, based on a cut-off of ≥ 13

Similarly to the results for emotional exhaustion, psychological distress peaked at the spring 2021 measurement point in most groups (Fig. 2). Repeated measures ANOVA of psychological distress for participants for whom there were no missing data over four time-points (77 nurses, 103 other healthcare professionals, 60 other clinical staff, and 110 non-clinical staff) demonstrated significant between-subject effects of occupational role (F = 3.6, *p* = .01) and age (F = 33.1, *p* < .001), but not ethnic group (F = 1.0, *p* = .33). The interaction of occupation role X time was not significant (F = 0.6, *p* = .83), indicating a similar pattern of change over time in all groups.

Post hoc testing revealed that the relationship between age and psychological distress was inverse (higher psychological distress with younger age) and significant at each time point (T1 R = −.24, T2 R = −.29, T3 R = −.24, T4 R = −.29, *p* < .001 at each time point). The proportion of participants with high psychological distress at each time point, by occupational role, is illustrated in Fig. 2. The severity of psychological distress was highest in nurses.

## Discussion

**Fig. 3:**
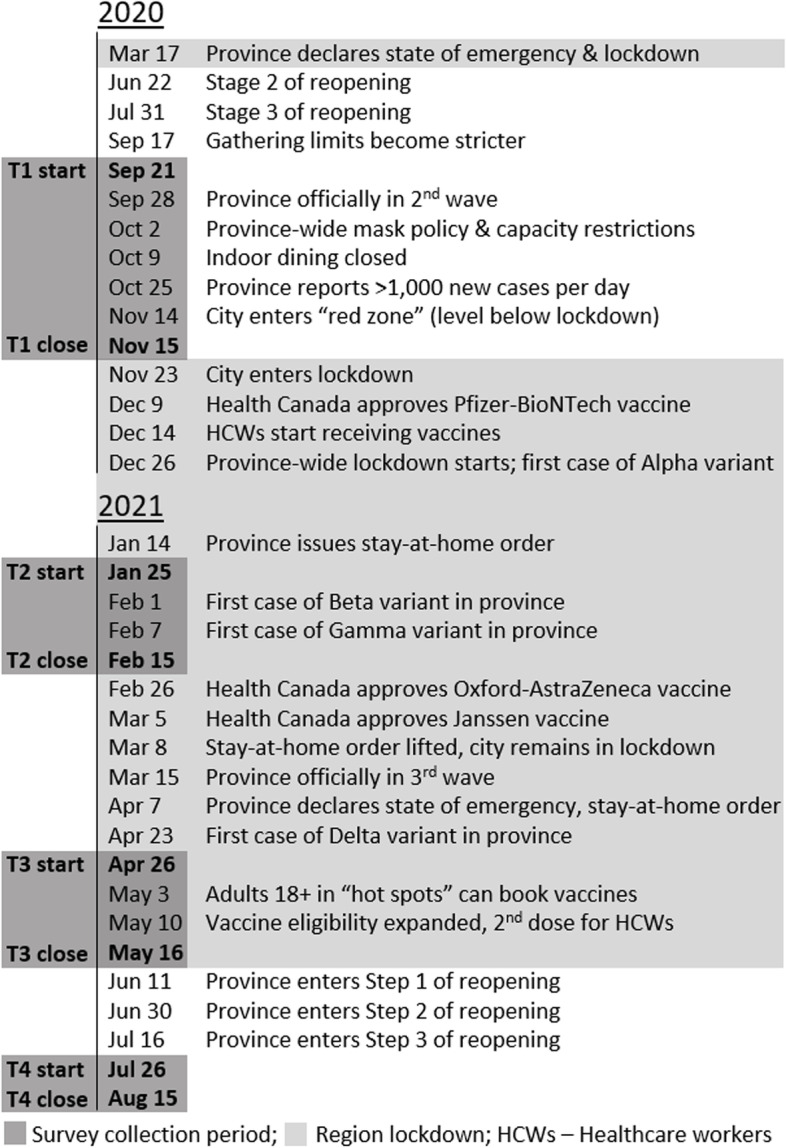
Timeline of major pandemic-related events in Toronto during the study

In this longitudinal study of a single cohort of hospital-based health care workers over approximately 1 year during the COVID-19 pandemic, negative mental health indicators measured at 3-month intervals changed significantly over time and varied between workers with different occupational roles. In order to appreciate the relationship of these temporal changes in healthcare worker well-being to dynamic fluctuations in pandemic-related stressors at work and in the community, we have provided local epidemiological curves in Figs. 1 and 2, and a timeline of major events in Fig. 3.

The severity of emotional exhaustion was greatest in nurses, followed by healthcare professionals of other disciplines, then nonprofessional staff whose work involves close patient contact, with the lowest levels in nonprofessional staff whose work does not involve close patient contact. While workplace factors that contributed to burnout prior to the pandemic, including high workload, lack of scheduling flexibility, and concern about workplace safety [41], have increased during the pandemic for all health professionals, arguably some stressors, such as persistent exposure to high acuity or dying patients, and facilitating patient-families meetings via tablets or other virtual interfaces have been more common for nurses, which could contribute to this difference. In addition, at the hospital at which this study took place, redeployment to critical care units [23], and the experience of working a full shift on a unit under “outbreak status” were more common for nurses than other professions. Pandemic conditions have led to moral distress, which exacerbates burnout [21, 22], but it is not known if this has been disproportionality experienced by particular occupational groups. Although not surprising, rates of high emotional exhaustion in nurses are nonetheless alarming. At the four measurement-points they were 54, 62, 63, and 58% respectively, which is substantially greater than the pre-COVID-19 benchmark of 20–40% found using the same operational case definition [29]. As nurses represent the largest single professional group in hospital care, this degree of mental health burden threatens the function of the healthcare system.

The rates of burnout in this study can be compared to those reported elsewhere, although most reports currently available relate to studies completed before 2021. In August – October 2020 a survey of Australian nurses using an abbreviated version of the MBI found high emotional exhaustion scores in 44% [42]. A US study conducted over the same time span using a single item burnout measure found high burnout in 66% of nurses [43]. A study of nurses in Uganda in May–June 2021 using the ProQOL instrument found high burnout in 40% [44]. The use of different measurement instruments precludes direct comparison, but these studies confirm multiple reports of high rates of burnout in nurses. A survey of internists at two hospitals in Vancouver, Canada from August–October 2020, found high emotional exhaustion in 63% using the same operational definition used in this study [45], which is consistent with our results. Burnout has not often been measured previously in hospital employees with roles other than regulated professionals, and so there is little historical context from which to interpret the degree of burnout found in that group.

The temporal pattern of change in emotional exhaustion differed very little between occupational groups. Inspection of Fig. 1 indicates that in three of the four groups this pattern consisted of a monotonic increase from fall 2020 to spring 2021, with a decrease from spring to summer 2021. The fourth group, nonprofessional staff with no close patient contact, appeared to differ only by beginning the descending trend sooner, peaking in winter 2021. Since the decrease in rates of high emotional exhaustion in summer 2021 corresponds to a period of low levels of new COVID-19 cases in the community, and corresponding low rates of COVID-19 related hospitalization, the trend may indicate that emotional exhaustion in hospital-based healthcare workers is able to recover somewhat as COVID-19 stresses subside. It is noteworthy that COVID-19 vaccination rates in the general public and in healthcare workers increased markedly in Ontario between the spring and summer 2021 surveys, which may have also contributed to reduced burnout. Importantly, the improvement in high emotional exhaustion during the summer of 2021 was an improvement to levels that remain much higher than the pre-COVID-19 benchmark.

Rates of high depersonalization and low personal accomplishment observed in this study are also concerning. Rates of depersonalization increased steadily over this time, which may indicate that depersonalization is a cumulative effect of pandemic related stress that is less responsive to decreasing case rates than emotional exhaustion. Alternatively, depersonalization could be viewed as a coping strategy (i.e. sacrificing empathic patient care in the service of maintaining personal function). Further observation to determine the down-stream consequences of these changes would help distinguish whether increasing depersonalization provides any adaptive benefit. Our participants’ sense of personal accomplishment may have been quicker to recover. Alternatively, the improvement in mean levels of personal accomplishment from winter 2021 to spring 2021 may have been the result of participants with low personal accomplishment scores in the winter 2021 dropping out. Post hoc testing reveals that at the winter 2021 measurement, the mean personal accomplishment scores of those who completed surveys in spring 2021 was 6.0 ± 7.0, whereas the mean score in those who did not complete surveys in spring 2021 was 4.3 ± 4.9 (*p* = .03). Of note, dropping out from the survey cohort could be related to factors which also contribute to staff choosing to leave their jobs. The risk that pandemic-related burnout will contribute to workforce shortages is an important issue [41] and the role of personal accomplishment merits further investigation as a potential mediator.

Previous longitudinal studies of burnout during the pandemic have used different measures (including single-item measures) which reduces the utility of comparing scores, but with respect to longitudinal trends studies have reported an increase from pre-pandemic to early-pandemic levels in intensive care settings [21] and an increase from April/May 2020 to July/August 2020 in oncology professionals [46]. Two studies have reported multiple serial measurements of burnout over a period of approximately 1 month. Four measurements of burnout at weekly intervals in April 2020, revealed stable levels of burnout in emergency medicine providers [47]. In contrast, burnout measured in physicians at five time-points over 25–31 days (following joining the COVID-19 treatment team) in Italy revealed variation over time [48]. The results of the current study, which indicate variability over time, rather than monotonic changes in emotional exhaustion and personal accomplishment provide new insight into the temporal pattern of burnout when it is measured over more than two time-points and for longer than a few weeks.

Psychological distress also changed over time in these hospital-based workers. As with emotional exhaustion, psychological distress was higher in nurses than in other healthcare professionals at each time point. Rates of psychological distress above the cut-off used in this study are clinically meaningful, because they indicate likely serious mental illness [37]. Thus, it is concerning that the case rate of high psychological distress was 10.2, 17.2, 21.3, and 13.1% in nurses at the four measurement points. Although temporal patterns of psychological distress appeared more variable than the patterns of burnout, inspection of Fig. 2 indicates that psychological distress was not monotonically rising in any occupational group.

The levels and patterns of emotional exhaustion and psychological distress measured in this hospital-based cohort raise concern for both the well-being of hospital-based healthcare workers and for the resilience of the healthcare system. Broad surveys of healthcare professionals in the same region during the COVID-19 pandemic indicate not only high rates of stress and burnout, but also that 43% of nurses surveyed were considering leaving healthcare work, and that this consideration was linked to feelings of high stress [49, 50]. While in other contexts the intention to leave one’s profession does not necessarily translate into action, multiple media reports of emergency department closures, cancelled surgeries due to understaffing, and a “signing bonus” for new emergency department nursing hires, indicate that pandemic-related workforce shortages are emerging [51–55]. This may be due to a vicious cycle of workplace conditions in which understaffing and increased workload are both a cause and consequence of high levels of emotional exhaustion, depersonalization, and psychological distress, as workers choose other alternatives [41].

Recognition of the mental health costs of healthcare work during the pandemic, and the related threat to the healthcare workforce, have led to recommendations for interventions to support healthcare workers that can be implemented at the level of healthcare systems and organizations, supplemented by individual [41]. Recommended interventions have included limiting shift lengths, maximizing scheduling flexibility, ensuring adequate training for unfamiliar tasks, providing support for moral distress, and promoting effective authentic leadership [41].

Our study’s finding that lower age and, by implication, less experience in healthcare, is associated with greater emotional exhaustion and psychological distress is consistent with numerous previous studies [27, 39, 56, 57]. It is also relevant that younger adults may be more likely to have children at home, which is known to have added stress during the pandemic [33]. Special efforts to support trainees and new graduates may include transition to practice programs that include formal teaching and mentorship over several months [58]. This consideration is especially relevant because widespread healthcare workforce shortages may lead to increases in training positions for healthcare professionals in order to increase the size of the pool of workers, leading to a younger healthcare workforce and a relative loss of senior mentors.

Our finding that rates of both burnout and psychological distress rise and fall in a pattern that is possibly attributable to changes in community-level and organization-level stressors is a pattern that suggests healthcare workers are resilient in spite of substantial adversity. Further research is required to identify factors that may promote faster and more complete recovery.

The strengths of this study include its prospective measurement of psychological distress and burnout with validated instruments over a one-year period in a single cohort of healthcare workers and its relatively high retention rate considering the extraordinary circumstances of hospital work during this time. It is also a strength that the survey extended to all workers and learners in the hospital, not just those in regulated professions. Although we consider retaining 76% of participants over four survey waves in 1 year to be a strength, the loss of 24% of the cohort nonetheless introduces the possibility that non-participation results in biases. Several aspects of the study may limit generalizability including that it did not using a sampling strategy that would ensure a representative sample, evidence that psychological indices (i.e. burnout) differ in participants who completed all four surveys and those who did not, its setting in an urban teaching hospital, and high rates of vaccination among healthcare workers after the second survey. Despite enrolling participants based on their willingness to participate, the distribution of occupations and demographic measures suggest that most occupational groups are represented among the survey’s participants. A further limitation is that classifying heterogeneous occupational roles into four occupational groups does not attend to differences in the impact of the pandemic between roles that are sorted together.

## Conclusions

In this cohort, neither emotional exhaustion nor psychological distress was rising monotonically, which provides hope that the negative mental health consequences of working in healthcare during the COVID-19 pandemic may improve as occupational stressors diminish. The depersonalization dimension of burnout, however, was found to rise consistently from one time point to the next. Burnout and psychological distress were consistently related to occupational role and were highest in nurses. Although emotional exhaustion improved as the case rate of COVID-19 decreased, rates of high emotional exhaustion remained much higher than was typically measured in hospital-based healthcare workers prior to the pandemic.

These results indicate that ongoing monitoring of healthcare workers’ mental health is warranted to determine the rate of recovery of burnout and distress as the COVID-19 pandemic recedes. Attention to organizational and individual interventions to support healthcare workers to maintain the resilience of the health care system continues to be important.

## Data Availability

The datasets used and/or analyzed during the current study are available from the corresponding author on reasonable request.

## Abbreviations

ANOVA: Analysis of variance
COVID-19: Coronavirus disease
